# (In)coherence between Chagas disease policy and the experiences of those affected in Mexico: The need for a transdisciplinary approach

**DOI:** 10.1371/journal.pntd.0013052

**Published:** 2025-05-07

**Authors:** Mariela Aké-Chan, Mariana Sanmartino, María Teresa Castillo-Burguete, Adriana González-Martínez, Carlos N. Ibarra-Cerdeña

**Affiliations:** 1 Departamento de Ecología Humana, Cinvestav, Mérida, México; 2 Consejo Nacional de Investigaciones Científicas y Técnicas, Instituto de Física de Líquidos y Sistemas Biológicos, Grupo de Didáctica de las Ciencias, La Plata, Buenos Aires, Argentina; 3 Departamento de Investigación, Salvando Latidos A.C., Guadalajara, Mexico; 4 Departamento de Investigación, Instituto Cardiovascular de Mínima Invasión (ICMI), Hospital Puerta de Hierro Andares, Guadalajara, México; RTI International, UNITED STATES OF AMERICA

## Abstract

Chagas disease, caused by the parasite *Trypanosoma cruzi*, remains a significant public health challenge in México, symbolizing systemic neglect in healthcare. Despite longstanding efforts to control its transmission, there are critical gaps in the alignment of public health policies with the lived experiences of affected individuals. This study examines these dissonances by analyzing qualitative interviews with 61 individuals diagnosed with *T. cruzi* and reviewing relevant Mexican public health regulations, including national standards and action programs. Findings reveal that most diagnoses occur incidentally, such as during blood donation or vector control campaigns, with minimal active case detection at the primary healthcare level. Affected individuals often encounter insufficient follow-up care, significant barriers to treatment, and misinformation that exacerbates psychological distress. Among the 14 participants who received etiological treatment, access was frequently due to individual persistence rather than systemic support, highlighting inequities in healthcare delivery. Additionally, structural barriers, including economic constraints and insufficient local healthcare infrastructure, further limit access to timely diagnosis and treatment, particularly in rural areas. Policy gaps include the absence of universal *T. cruzi* testing for pregnant individuals, lack of vertical transmission prevention strategies, and inadequate communication between healthcare providers and patients. Current public health initiatives disproportionately prioritize vector control and blood bank screening, neglecting the broader social and economic challenges faced by those already diagnosed. The study underscores the urgent need for a transdisciplinary approach to Chagas disease management in México, integrating biomedical, sociocultural, and policy perspectives. Recommendations include implementing universal prenatal screening for *T. cruzi*, enhancing health communication strategies, reframing Chagas as a manageable condition to reduce stigma, and improving follow-up care protocols. Addressing these challenges requires intersectoral collaboration and an inclusive approach that values the lived experiences of affected communities. By bridging the gap between policy and practice, this research contributes to the development of holistic strategies that not only control Chagas disease transmission but also improve the quality of life for those already impacted. These insights are essential for informing public health reforms in México and other endemic regions, advancing equity and effectiveness in neglected tropical disease management.

## Background

From a biomedical perspective, Chagas disease is an infection caused by the parasite *Trypanosoma cruzi* [1]. However, Chagas disease is more than just an infection or illness. It symbolizes neglect in public health. Despite its discovery in 1909, significant knowledge gaps persist, ranging from the accurate global count of infected individuals to the incidence of the disease. Most concerning is the lack of awareness about the issue among the general public and healthcare personnel [2–4]. *T. cruzi* is transmitted via the feces of vector insects (Hemiptera: Reduviidae), during gestation or childbirth from infected individuals, through blood transfusion, organ transplantation, or orally. The infection progresses through two phases: an acute phase, lasting 4–8 weeks, and a chronic phase, in which approximately 30% of infected individuals present symptoms, while the remaining 70% remain asymptomatic [1]. Currently, only two medications effective against the parasite exist, both developed over half a century ago. These drugs are particularly effective in young individuals or when administered during the acute phase [5].

For decades, international organizations and governments have developed and promoted strategies to control Chagas disease transmission, focusing primarily on interrupting transmission through blood transfusions and controlling or eliminating domestic vector populations [6–8]. While the strategies to eliminate transfusion and transplant-related transmission have been largely successful, vector control in endemic countries continues to face significant challenges, including insecticide resistance and reduced efficacy [9]. Regarding vertical transmission, its prevalence may be underestimated, as routine screening for infection in individuals with the capacity to gestate is not included in prenatal care in several countries [10]. In response, PAHO incorporated Chagas disease into its 2017 framework for the elimination of mother-to-child transmission of selected diseases. However, to date, countries such as Mexico have not adopted this strategy [10].

In addition, barriers to diagnosis and treatment remain substantial and widely acknowledged, with agreements in place to address these obstacles [6–8]. As Tarleton et al. (2014) observed, achieving these goals requires not only ambitious objectives but also adequate investment and sustained political commitment to strengthen health systems. Efforts must extend beyond theoretical frameworks of official standards, regulations, plans, and strategies to produce tangible results [9].

Furthermore, researchers emphasize the importance of integrating a sociocultural approach that considers the lived experiences of individuals affected by *T. cruzi* infection. These individuals are directly impacted by policies and interventions, making it essential to include their perspectives in the understanding, development, and implementation of strategies [11–14].

Social research perspectives, however, are often underrepresented in research priorities, such as those outlined by the TDR Disease Reference Group for Chagas Disease, Human African Trypanosomiasis, and Leishmaniasis [8]. These priorities predominantly focus on advancing diagnostic methods, therapeutic medications, and insecticide technologies, which are undeniably critical for disease control. Nevertheless, there is an opportunity to further integrate approaches that address social inequalities, barriers to healthcare access, and the broader implementation of public health interventions in diverse contexts. In contrast, other TDR initiatives, such as the Disease Reference Group on Zoonoses and Marginalized Infectious Diseases of Poverty [15], emphasize community-based approaches and social research frameworks. These initiatives demonstrate the importance of addressing the needs of marginalized populations who have limited or no access to governmental health services, illustrating the value of integrating social perspectives to create more inclusive and impactful health interventions. Strengthening this dimension would complement technological advancements and foster the development of more holistic strategies to fight Chagas.

One of the most significant projects aimed at interrupting vector and transfusion transmission of *T. cruzi* is the Central American Countries Initiative (IPCA), which was established in 1990. Mexico joined this initiative in 2013, expanding it to IPCAM (Central American Countries and Mexico Initiative). This marked a pivotal moment, signaling Mexico’s international commitment to addressing Chagas disease [16]. However, the country has been characterized as “passive” in actively seeking and diagnosing infected individuals [9,17].

## Chagas disease in Mexico

In 1938, the first two cases of *T. cruzi* infection were identified in Mexico [18]. However, it was not until 2012 that the notification of cases and associated control and prevention activities became mandatory. This change occurred with the inclusion of Chagas disease in the official Mexican standard, NOM-032-SSA2–2014, which covers the epidemiological surveillance, promotion, prevention, and control of vector-borne diseases [19]. In the same year, mandatory screening for *T. cruzi* in blood banks was established through NOM-253-SSA1-2012, regulating the use of human blood for therapeutic purposes [20]. Before this regulation, the prevalence of *T. cruzi* in blood banks ranged from 0.3% to 17.5%, higher than the prevalence of HIV and hepatitis B [21].

In 2013, the National Center for Preventive Programs and Disease Control (CENAPRECE), a division of the Ministry of Health responsible for implementing preventive programs, launched the first Specific Action Program (PAE) to address Chagas disease. Annex 7 of the PAE (2013–2018) presents data showing that, although the percentage of blood samples screened for *T. cruzi* increased from 13.19% in 2001 to 90.6% in 2012, Mexico had not yet reached full (100%) coverage by that time. According to this report, which analyzed data up to 2012, Mexico was the only country in the Americas that had not achieved universal screening of blood donors [22,23]. Moreover, blood bank screening depends on the availability of reagents, meaning that not all samples are consistently tested [17]. Another issue is the potential for antibody cross-reactivity, making the specificity and sensitivity of testing kits a key selection criterion [24]. However, in Mexico, the types of kits used remain unknown [17].

The last National Seroepidemiological Survey was conducted in the late 1980s and reported a prevalence of 1.5% [25]. In 2015, the World Health Organization (WHO) estimated that 876,458 people were infected with *T. cruzi* in Mexico [26], while Arnal et al. (2019) calculated the figure to be approximately 4 million [27].

According to the Ministry of Health, positive *T. cruzi* cases are reported weekly in the National Epidemiological Bulletin, published by the General Directorate of Epidemiology (DGE). However, the 2021 PAE indicates that, as of 2019, vector-borne disease surveillance faced significant resource and budgetary challenges. Most of the budget (96.3%) was allocated to dengue and malaria, with only 3.7% reserved for managing four other conditions, including Chagas [28]. This funding disparity reflects the limitations in health teams’ capacity to report cases, raising questions about the accuracy of the data in the epidemiological bulletin.

Academic research on Chagas disease in Mexico dates back to the mid-20th century [29]. However, vector-related studies dominate the literature (>140 publications), while social aspects remain underexplored, with only nine publications in the last decade. This disproportion is not unique to Mexico. While the country ranks seventh in Chagas-related academic production, with Brazil leading [30], research in Brazil is overwhelmingly clinical, with no social science studies identified in a review of publications from 2012 to 2016 [31]). Thus, although Mexico publishes less than Brazil or Argentina, the global trend highlights the persistent gap in qualitative and social science research on Chagas. Our study contributes to addressing this gap by advocating for a more diversified research approach, particularly in socio-ecological perspectives and community engagement in vector control. Moreover, academic and community knowledge has yet to be integrated into public health programs [29]. To the best of our knowledge, no scientific studies have documented the experiences and knowledge of affected individuals in Mexico, particularly their efforts to mitigate the consequences of infection. This research provides an opportunity to deepen our understanding of Chagas disease from the perspective of those living with it and to examine how national policies align with the realities of those directly affected.

## Methods

### Ethics statement

The research protocol was approved by the ethics committee of the Centro de Investigación y de Estudios Avanzados del Instituto Politécnico Nacional (Cinvestav) (reference: 087/2022). Written informed consent was obtained from all participants.

### Case interviews

**Fig 1:**
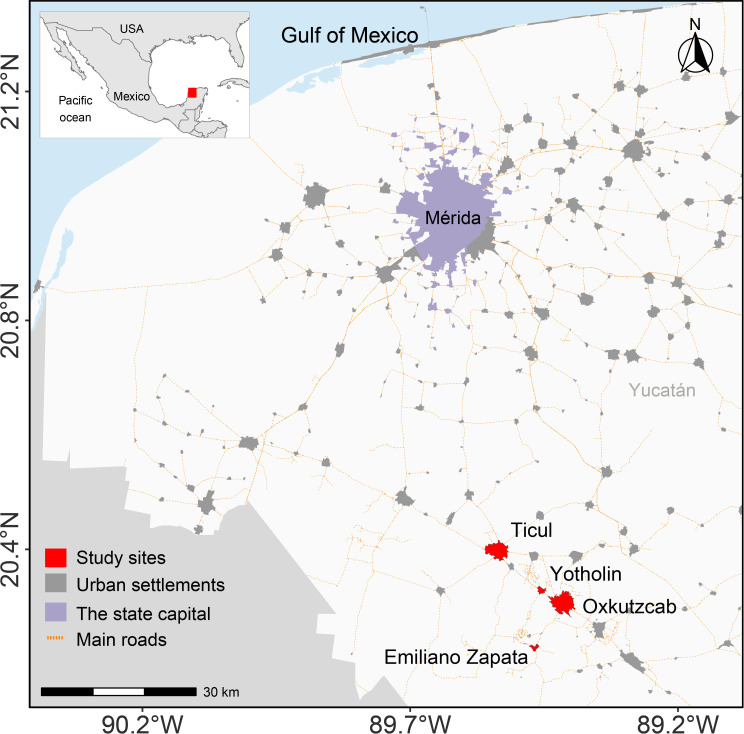
Study area. The base map was generated using R software v4.0.1 (https://www.r-project.org/) and publicly available data on urban settlements, major roads, and national boundaries of Mexico, provided by the National Institute of Statistics and Geography (INEGI; https://www.inegi.org.mx/app/mapas/; accessed February 14, 2025). Terms of use are available at https://en.www.inegi.org.mx/inegi/terminos.html. International boundaries were obtained from Natural Earth, with all maps in the public domain (http://www.naturalearthdata.com/about/terms-of-use/).

The field study was conducted in the state of Yucatán, located in southeastern Mexico, one of the three states with the highest incidence of *T. cruzi* infection. The southern municipalities of Yucatán are classified as highly endemic areas, reporting the highest numbers of *T. cruzi* cases in humans. [28,32]. This study focused on four communities within this region: Emiliano Zapata, Oxkutzcab, Yotholín, and Ticul (Fig 1). Despite their geographical proximity, these communities exhibit structural disparities in access to communication, transportation, and healthcare services. Proximity to Mérida, the state capital, correlates with greater availability of healthcare services, as Mérida hosts the majority of second-level public hospitals and the state’s only third-level public hospital.

Using a database compiled from records provided by the Ministry of Health and previous studies conducted by some of the co-authors (CNIC and AGM), individuals diagnosed with *T. cruzi* were identified and invited to participate. A total of 58 individuals agreed to participate. Additionally, three individuals not listed in the database were included in the study after being recommended by existing participants. The participants represented four communities: Emiliano Zapata (female = 3; male = 8), Oxkutzcab (-female = 8; male = 8), Yotholín (female = 5; male = 5), and Ticul (female = 1; male = 26). Information about participants, including symptomatic status, the institution responsible for the diagnosis, etiological treatment, following diagnosis, community, age, employment status, sex, education level, and language (i.e., whether they remain in the asymptomatic stage of the disease), is available in the supplementary table (S1 Table).

Between October 2022 and March 2025, 61 semi-structured interviews were conducted. These interviews addressed four thematic areas adapted from Cuevas (2016): sociodemographic data, knowledge fields, production, and attitudes [33]. Key questions included: “When did you first hear about Chagas disease?” “Who did you discuss it with?” and “What were you told?” Based on these prompts, participants narrated their stories, including their experiences with diagnosis, treatment, and follow-up care.

### Review of official documents

A comprehensive review of official documents regulating actions related to institutional care for Chagas disease in the country was conducted. The reviewed documents are summarized in Table 1. These documents are organized hierarchically, with Mexican Official Standards (Normas Oficiales Mexicanas, NOMs) occupying the highest level, followed by the specific action program. Manuals and guidelines, which serve as technical resources to facilitate the implementation of strategies, are positioned at the lowest level.

**Table 1:** Official documents regulating actions related to institutional care for Chagas in Mexico.

Official documents	Scope	Goal	Date of publication
1. NOM-032-SSA2–2014^*^	Epidemiological surveillance, promotion, prevention, and control of vector-borne diseases.	Establish specifications, criteria, and procedures aimed at reducing the risk of infection, illness, complications, or death caused by diseases transmitted by vectors.	In the Official Journal of the Federation on April 16, 2015
2. NOM-017-SSA2–2012^*^	Epidemiological surveillance, establishes the criteria, specifications, and operational guidelines for the National Epidemiological Surveillance System (SINAVE).	Systematically, continuously, promptly, and reliably collect relevant and necessary information about the population’s health conditions and their determinants.	In the Official Journal of the Federation on February 19, 2013.
3. NOM-253-SSA1–2012^*^	Disposition of human blood and its components for therapeutic purposes, establishes the activities, criteria, strategies, and operational techniques concerning the collection, processing, storage, distribution, and clinical use of human blood and its components.	Ensure the safety, quality, and efficacy of blood and blood components used for therapeutic purposes, thereby protecting the health of both donors and recipients.	In the Official Journal of the Federation, 26 October 2012. It was officially modified on December 22, 2021, and a new draft dated November 8, 2024, is available.
4. Laboratory Guidelines for Chagas Disease^**^	Comprehensive protocols for the diagnosis, treatment, and management of Chagas disease. These guidelines focus on accurate detection of *Trypanosoma cruzi*, through various diagnostic methods, including serological and molecular techniques.	Standardizing laboratory practices in Mexican health centers.	In March 2019 by the Centro Nacional de Programas Preventivos y Control de Enfermedades (CENAPRECE), a branch of Mexico’s Secretaría de Salud
5. Standardized Procedures Manual for the Epidemiological Surveillance of Vector-Borne Diseases (MPETV)^**^	Comprehensive guide developed to standardize and enhance the surveillance of vector-borne diseases in Mexico.	Establish standardized protocols for detecting and monitoring vector-borne diseases, provide guidelines for efficient data management, and enhance coordination among health agencies to improve disease prevention and control efforts.	2021
6. Specific Action Program for Vector-Borne Diseases (PAE)^**^	Strategic initiative of the Mexican Ministry of Health to reduce the incidence and impact of vector-borne diseases.	Strengthening epidemiological surveillance, implementing integrated vector control strategies, promoting community awareness and participation, and coordinating intersectoral actions to address environmental and social factors driving vector-borne diseases.	2021

*Compulsory throughout the national territory for health service personnel in all sectors of the National Health System.

**Not mandatory, supplementary documents produced by the health authorities themselves as support for standards.

### Data analysis

Common themes were identified through the analysis of documents and interviews using a qualitative approach known as open and axial coding [34]. This process facilitated the development of categories to assess whether the management prescribed in Mexican policies aligns with the contextual needs and challenges faced by affected individuals. The coding process was conducted using ATLAS.ti software version 9.0. This analysis yielded four primary categories: *T. cruzi* diagnosis, etiological treatment, diagnostic follow-up, and parasite transmission control.

## Results

### Diagnosis of *T. cruzi*

According to the Mexican Official Standard NOM-032-SSA2–2014, an individual is considered to have Chagas disease when the presence of the *T. cruzi* parasite is confirmed. In the acute phase, diagnosis is confirmed by direct observation of the parasite (thick or thin blood smear microscopy), the Strout concentration technique, PCR, culture, subinoculation of blood, and/or positive serology (ELISA, IFI, or HAI) starting four weeks post-infection. In the chronic phase, with or without symptoms, clinical diagnosis is confirmed through positive serology (ELISA and IFI or HAI) and/or parasitological diagnosis (thick or thin blood smear microscopy, PCR, subinoculation, indirect xenodiagnosis, and hemoculture).

For the application of the aforementioned diagnostic tests, the MPETV establishes that presumptive diagnosis responsibilities fall under local-level healthcare facilities, such as Health Centers and Hospital Units. These facilities are responsible for taking samples to confirm the presence of *T. cruzi* operational definitions of signs and symptoms described in the MPETV as well as the association of epidemiological evidence, like residence in an endemic area [35].

### Experiences with diagnosis

Out of the participants interviewed, 57 individuals learned of their *T. cruzi* positivity incidentally, either during blood donation or as part of a -research study or a vector campaign conducted in their locality. In only four cases did healthcare personnel suspect potential infection, request relevant tests, and provide a diagnosis.

One of these cases involved a woman whose symptoms baffled her cardiologist until further investigations led to a *T. cruzi* diagnosis:

*“The doctor, a cardiologist treating me, decided to investigate further and said, ‘You know what? We’re going to do these Chagas tests.’ Boom! It came back positive.”*

*(Female, symptomatic, 42 years old)*

Another case involved a young man whose father, working as a health institution driver, recognized symptoms indicative of *T. cruzi* infection. Without his father’s intervention, the young man acknowledged he would not have sought medical attention. The third and fourth cases involved a woman and a man who presented with a chagoma. The woman sought medical care due to her knowledge of Chagas transmission, while the man consulted a physician due to the painful manifestation on his skin.

In these cases, directed diagnosis at the primary healthcare level was non-existent. If the goal is to achieve timely detection, what should be the most appropriate criteria for active case finding? Since most individuals were diagnosed incidentally, such as during blood donation, this raises significant questions.

### Insights from blood donation diagnostics

For 62% of participants, learning of their *T. cruzi* positivity occurred during blood donation. The following excerpt summarizes their experiences:

*“My mother was ill, so they drew my blood. I’d donated four times over two years, but on the fifth time, the nurse told me, ‘Your blood doesn’t pass; you have Chagas markers.’ I asked, ‘What’s that?’ She explained briefly: ‘It’s a bug that bit you in the forest, your house, or your farm.’”*

*(Male, 41 years old, asymptomatic)*

For 45 individuals, this brief and often alarming explanation marked their first encounter with the term “Chagas.” One participant shared their experience of being told they only had five years to live:

*“They told me I had five years left to live; that I’d have a heart attack and die.”*

*(Male, 36 years old, asymptomatic)*

The information provided by hospital staff can, in some cases, trigger a state of resignation, leading individuals to choose not to seek further information, neglect their health, and simply wait for death. We identified this situation in 12 out of 61 participants, in whom resignation was the dominant sentiment. Notably, none of these 12 individuals received treatment or medical follow-up, despite at least two women in this group being symptomatic. When asked whether they would like more information about Chagas disease, participants expressed confusion and fear, as illustrated in the following example.

*“Well, yes and no—because I do know I have it, right? And knowing I have it does make me curious about what might happen to me. But also no, because if I know, I feel like it’ll become a psychological thing. Like I told you, I already went, I already tried, and nothing—no one has said, ‘Hey, we’re going to run some tests’ or ‘We’ll follow up.’ So with the kind of job I have and the income I earn, I won’t be able to afford treatment and all that. I’d rather a thousand times shut down the possibilities of what might happen, be ignorant about it, because, like I said, it would just make me scared. I have three kids, I have my family, and I know that if something happens to me… living with that fear, I mean, what for?”*

*(Female, 36 years old, asymptomatic).*

### Follow-up to diagnosis

The NOM-032-SSA2-2014 mentions: *“It is recommended that all patients carrying* Trypanosoma cruzi*, regardless of their disease stage, undergo evaluation to receive etiological and/or symptomatic treatment according to their clinical presentation. Additionally, blood donation by these individuals should be discouraged.”* Section 7.3.3.1 recommends providing treatment to patients up to 70 years old who have tested positive in reference laboratories, both in acute and chronic stages of less than two years of progression. This includes recent recipients of accidental infection through blood transfusion or transplantation, reactivations of chronic infections due to various types of immunosuppression, and newborns with a confirmed diagnosis of congenital infection. In chronic infections, treatment is optional and subject to medical-patient evaluation, considering the limited potential outcomes and the toxicity of the medication. In section 7.3.3.2, it is stated that symptomatic patients, regardless of their disease stage, should be evaluated for receiving etiological and/or symptomatic treatment according to their clinical manifestations and diagnostic findings. For the acute or indeterminate phase, section 7.3.3.3 recommends the use of nifurtimox, as it is highly effective in both the acute and chronic phases for asymptomatic patients under 18 years old. The section also specifies the dosage for administering benznidazole. Beyond the aforementioned recommendations, the standard does not provide guidance on managing side effects.

The next section in the standard outlines the parameters for evaluating treatment. It specifies that once etiological treatment is completed, individuals remain positive for the parasite: *“The case will be cataloged as active, not subject to further therapy with specific drugs, but requiring medical surveillance every five years. If symptoms attributable to T. cruzi infection arise, annual evaluations are recommended as determined by the treating physician.”* However, for individuals who are not candidates for treatment, the standard does not specify the procedures to follow.

In Mexico, etiological treatment is only provided by the Ministry of Health, and only for cases confirmed through tests conducted by laboratories belonging to the National Network of Public Health Laboratories (RNLSP). Notably, MPETV does not clarify which level of healthcare administration is responsible for delivering the treatment.

### Experiences following diagnosis

Among those interviewed, 28 individuals were scheduled for follow-up appointments after their diagnosis. However, 12 of these individuals stopped attending due to various reasons: they never received treatment, their questions went unanswered, they were seen by multiple physicians who provided contradictory explanations, or their follow-up was disrupted by the SARS-CoV-2 pandemic:

*“They detected it in 2011, the year my dad passed away. I followed up for six years, but then I stopped because the pandemic hit, and there were no open clinics or consultations, so I never went back.”*

*(Female, asymptomatic, 62 years old)*

Of the remaining 33 individuals who did not receive a medical order for follow-up consultations, 14 reported sporadic visits by health personnel without any formalized or specific records. A 48-year-old symptomatic woman commented on these visits:

*“They come to check if I’m still alive (laughs)… I think they’re required to do reports so they know… they visit from time to time; they know who is positive and come to see us.”*

Nineteen participants never received a medical order to attend consultations or follow up on their diagnosis. Within this group, five individuals sought support independently, and two managed to obtain treatment through their persistence. One was a 59-year-old symptomatic man diagnosed during an academic research project. He sought out three different doctors across public and private healthcare institutions and eventually received treatment, though he never learned who authorized the medication:

*“To this day, I still don’t know who requested or sent the medication.”*

The other individual, a 55-year-old man, explained

*“I found out during blood donation when my wife needed surgery. I didn’t know anything about Chagas disease, but we started researching. My son works in healthcare, so he managed to get the medicine, and the health sector provided it.”*

### Gaps in treatment delivery

Only 14 individuals received etiological treatment. Of these, only two remembered the name of the medication, which was nifurtimox. Nine individuals had been diagnosed between 10 and 20 years ago and did not recall the name of the drug. Among those treated, some attributed their access to luck (male, 59 years old, symptomatic) or divine intervention (male, 55 years old, asymptomatic). Two individuals did not complete the treatment—one due to side effects and the other because it required frequent time off work to obtain the medication. Of the 12 who completed the treatment, none reported having follow-up medical appointments. Among the 46 individuals who did not receive treatment, 22 recalled unfulfilled promises of care from health authorities that had persisted for over a decade.

One unique case involved a 53-year-old man who participated in an event where electrocardiograms were conducted in a private home by individuals in medical attire, whom he believed to be doctors. The event also included a raffle for treatment. Despite being selected as a “winner,” he never received the promised medication. This event was mentioned by four other individuals from the same community, none of whom received documentation or remembered the names of the organizers or their institutions.

Some participants recalled being told by health professionals that treatment would not be provided due to various reasons, including:

- *“No abnormalities detected”* (male, 51 years old, asymptomatic)
- *“The treatment is like chemotherapy”* (female, 62 years old, asymptomatic)
- *“Not recommended for your age”* (male, 70 years old, asymptomatic)
- *“The medicine isn’t available in Mexico; it comes from Brazil or France*: *When they told me I had it, I started the process with my insurance, but eventually, they told me not to bother because it’s incurable. They said the treatment only exists in Brazil and isn’t available in Mexico.” (*male, 43 years old, asymptomatic*)*

### Structural barriers to follow-up

Healthcare access depends both on institutional capacity and individual willingness to attend appointments. However, personal willingness is conditioned by economic, social, and family factors. Most participants live day-to-day, and attending medical consultations often requires traveling to Mérida, incurring significant time and financial costs. One participant shared:

*“They told me I had Chagas, so I went to the health center, but all they did was check me, no medication. They scheduled me for appointments in Mérida every month or two. Eventually, I got fed up with the constant travel and expenses. Plus, I had to ask for permission from work, and my bosses would get annoyed.”*

*(Male, asymptomatic, 43 years old)*

### Control of T. cruzi transmission

The Specific Action Program (PAE) for 2021 proposes integrated vector management strategies in localities with confirmed cases of *T. cruzi* within the last three years and where the presence of *Triatoma barberi* and *Triatoma dimidiata* has been documented. Of the 94 localities targeted nationwide, 39% are in Yucatán. Vector control strategies include chemical and physical methods, as outlined in the NOM-032-SSA2–2014.

For controlling transfusion-related transmission, the strategy involves mandatory screening in blood banks as per NOM-253-SSA1–2012: *“All collected blood components must be tested for the detection of transmissible infectious agents such as the human immunodeficiency virus (HIV), hepatitis B and C viruses,* Trypanosoma cruzi, Treponema pallidum*, and others as circumstances may require”* [36]. To date, this approach has been the primary pathway for diagnosing Chagas disease.

Regarding vertical transmission (during pregnancy or childbirth), no specific prevention strategies are outlined in current regulations. The 2021 PAE mentions prioritizing work in communities with cases among children under 15 years old. However, it does not detail how individuals can access diagnostic services. Similarly, the MPETV specifies the necessary tests for case confirmation but provides no guidance on how these tests can be accessed.

### Experiences with vertical transmission and prevention

Among the participants, only one woman was diagnosed during her pregnancy, and this was through an academic research project. She was advised that nine months after her child’s birth, the baby should undergo testing; however, this follow-up never occurred:

*“The doctor diagnosed me during my pregnancy and told me I couldn’t receive treatment while pregnant. She wrote down the medication I’d need after giving birth and told me my baby should be tested nine months later. But that test was never done.”*

*(Female, 39 years old, asymptomatic)*

## Beyond reported cases

Public health policies traditionally evaluate their success through classical epidemiological measures, such as morbidity and mortality reports [37]. These reports depend heavily on planned strategies and government budgets. Their significance lies in their theoretical ability to provide quality epidemiological data to guide prevention and control actions [33]. However, numerical data alone cannot fully capture the unmet needs of affected populations.

**Fig 2:**
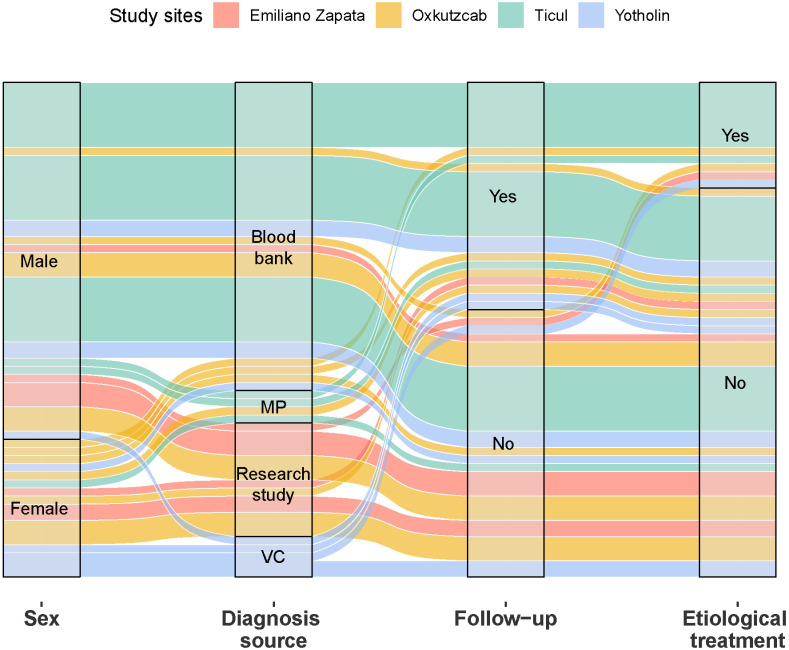
Alluvial diagram illustrating the distribution of individuals diagnosed with *T. cruzi* infection by sex, diagnostic source, follow-up, and etiological treatment. The diagram reveals complex interactions and transitions between categories within each variable, stratified by study site. This visualization facilitates the identification of patterns and associations between diagnostic, follow-up, and treatment processes, highlighting potential disparities in healthcare access and delivery across different localities. VC = Vector campaign, MP = Medical Practice.

This study revealed that the majority of participants were diagnosed incidentally, received inadequate treatment or follow-up, and remained uncertain about their condition, highlighting significant gaps in medical care (Fig 2). These findings reflect Mexico’s ongoing passive stance in the detection, diagnosis, and monitoring of Chagas disease. Notably, while at least one participant from each locality received treatment, the majority of those treated resided in Ticul, the largest urban settlement and closest to Mérida. This disparity in access to medical care and treatment appears to be largely influenced by urban-rural differences. Participants from remote areas faced unique challenges, including economic constraints due to the time and expense required to travel to urban centers for medical attention, as well as lost income due to unpaid leave from work.

The absence of people experiences and family perspectives in the production of knowledge about the disease has led to irreversible consequences and the failure of health intervention strategies [38,39]. Therefore, it is critical to promote collective knowledge construction and problem-solving efforts that avoid prioritizing one type of expertise over another. This inclusive approach can foster participation and commitment from all stakeholders [40].

## Recommendations for updating the regulations

Below, we present proposals derived from our findings. These recommendations aim to foster an intersectoral approach and pave the way for updates to public policies related to Chagas disease, both in Mexico and other affected countries.

### Universal testing for *T. cruzi* in pregnant individuals

We propose the inclusion of *T. cruzi* screening for all pregnant women, regardless of their socioeconomic status or place of origin, following the example of countries such as Argentina (2007) and Chile (2014) [41,42]. In line with this, PAHO launched an initiative in 2017 to eliminate mother-to-child transmission of Chagas disease and other vertically transmitted infections [10]. We emphasize the importance of Mexico adopting this strategy. Specifically, we recommend incorporating *T. cruzi* screening into NOM-007-SSA-2016, the national guideline that regulates care during pregnancy, childbirth, postpartum, and for newborns. This policy change could significantly improve health outcomes by enabling early detection and treatment, particularly for newborns with congenital *T. cruzi* infection, who have a high probability of being cured if treated promptly [5,43].

For individuals capable of pregnancy, providing treatment before conception can prevent vertical transmission in future pregnancies. It is important to note that etiological treatment is contraindicated during pregnancy and must be administered beforehand [44].

### Effective communication strategies

As demonstrated in this study and official guidelines, at present communication plays a minimal role in Chagas disease management. Most participants first learned they had Chagas disease during blood donation, with no prior awareness of the condition. \

To address this gap, we recommend:

1. Implementing communication strategies that clarify the objectives and procedures during blood donation processes.
2. Establishing health education programs for healthcare personnel at all levels, including blood bank staff, physicians, and nurses.
3. Developing communication workshops, guided by the principles of popular education, for healthcare staff. These workshops should encourage open dialogue, address doubts, and foster better understanding of diagnoses, thereby contributing to more effective and humane care.
4. It is essential to include a section in the official standard addressing communication between healthcare workers and patients to ensure that individuals fully understand their diagnosis, treatment options, and the necessary follow-up care. Healthcare professionals should provide clear, empathetic, and culturally sensitive explanations of diagnoses, ensuring that any concerns or misconceptions the people may have are adequately addressed.

### Reframing Chagas as a manageable condition

In the experiences shared, a Chagas diagnosis was often perceived as a death sentence, seemingly justified by the health system’s inability to address the condition adequately. However, this perception conflicts with the reality that only 3 out of 10 individuals will develop symptoms, and with regular annual check-ups, the disease can be managed effectively. This also reduces the economic burden of treatment [45].

Mexico’s shortcomings in communication and public awareness regarding Chagas disease present an opportunity to adopt precise and appropriate terminology. Drawing from studies conducted in other countries [12,46], and our findings, we advocate for reevaluating the use of terms such as “deadly disease” to describe Chagas.

### Improving follow-up guidelines

National guidelines must clearly outline follow-up steps for individuals diagnosed with *T. cruzi*, irrespective of whether they receive etiological treatment. Current prevention efforts in Mexico primarily aim to reduce new infections, but they fall short in addressing the needs of those already diagnosed.

Given that Chagas is a chronic infection, annual medical check-ups are essential to monitor potential parasite-related damage and provide timely interventions.

## Conclusion

Evaluating public health policies through the lens of those directly affected by Chagas disease highlights gaps and opportunities for improvement. This study reveals the profound disconnect between national policies and the lived experiences of affected individuals. Most participants were diagnosed incidentally, received no treatment or adequate follow-up, and remained uncertain about their condition. These deficiencies in medical care not only exacerbate the physical and psychological burden on individuals but also hinder broader efforts to control *T. cruzi* transmission.

We acknowledge that the experiences described in this article are influenced by the specific characteristics of the studied region, and therefore, it is not possible to generalize public policy management in Mexico to all regions of the country. However, at the national level, institutional and epidemiological barriers have been identified that hinder accurate knowledge of the number of infected individuals, leading to inadequate medical attention. In Mexico, Chagas remains a neglected public health issue, burdened by significant challenges at both institutional and community levels. For example, barriers such as the exclusion of antitrypanosomal medications from the national formulary, the absence of clinical guidelines, and limited awareness among healthcare providers significantly impede access to treatment and exacerbate the neglected status of infected individuals [47]. At the community level, insufficient understanding of the disease’s transmission dynamics and clinical manifestations undermines the prioritization of vector control measures and highlights the need for enhanced education and engagement in affected regions [48]. Additionally, a systematic review and meta-analysis revealed higher prevalence estimates than previously recognized, underscoring the urgent need to establish Chagas as a national public health priority to address its impact on Mexico’s most vulnerable populations [27]. These findings emphasize the importance of comprehensive strategies that integrate policy reform, healthcare provider education, and community engagement to address the multifaceted challenges posed by Chagas.

It is clear that current policies and strategies must evolve to integrate a sociocultural perspective that considers the diverse realities of those affected. Addressing structural barriers—such as economic disparities, limited access to healthcare, and insufficient public awareness—is imperative. Moreover, the health system must adopt a dialogical approach that values both scientific knowledge and lived experiences, fostering a collective effort to address the challenges of Chagas disease.

Achieving meaningful structural changes will require genuine intersectoral collaboration and the recognition that public health is a shared responsibility. By embracing these principles, we can move towards more inclusive, democratic, and sustainable solutions for Chagas and other neglected tropical diseases.

## Supporting information

S1 Table Summary of participant characteristics, including symptomatic status, institution responsible for diagnosis, etiological treatment received following diagnosis, community of residence, age, employment status, sex, education level, and primary language. (XLSX)
